# The Incidence, Risk Factors and In-Hospital Mortality of Acute Kidney Injury in Patients After Surgery for Acute Type A Aortic Dissection: A Single-Center Retrospective Analysis of 335 Patients

**DOI:** 10.3389/fmed.2020.557044

**Published:** 2020-10-15

**Authors:** Linji Li, Jiaojiao Zhou, Xuechao Hao, Weiyi Zhang, Deshui Yu, Ying Xie, Jun Gu, Tao Zhu

**Affiliations:** ^1^Department of Anesthesiology, West China Hospital, Sichuan University & The Research Units of West China (2018RU012), Chinese Academy of Medical Sciences, Chengdu, China; ^2^Department of Anesthesiology, Nanchong Central Hospital, The Second Clinical Medical College, North Sichuan Medical College, Nanchong, China; ^3^Division of Ultrasound, West China Hospital, Sichuan University, Chengdu, China; ^4^Department of Anesthesiology, The Second People's Hospital of Yibin, Yibin, China; ^5^Department of Cardiovascular Surgery, West China Hospital, Sichuan University, Chengdu, China

**Keywords:** acute kidney injury (AKI), acute type A aortic dissection (AAAD), kidney disease: improving global outcomes (KDIGO) criteria, risk factor, in-hospital mortality

## Abstract

**Background:** Acute kidney injury (AKI) is a common complication of cardiac surgery, which could lead to increased morbidity and mortality. Acute type A aortic dissection (AAAD) is a life-threatening cardiac disease and can be closely related to post-operative AKI. However, data on the incidence of AKI defined by the newest Kidney Disease: Improving Global Outcomes (KDIGO) criteria and in-hospital mortality of a homogeneous population who underwent AAAD are limited. We aimed to investigate the incidence of AKI defined by the KDIGO criteria and the risk factors associated with the outcomes among AAAD-induced AKI patients.

**Methods:** We reviewed 335 patients who underwent surgical treatment for AAAD between March 2009 and June 2016. We screened the patients' AKI status and analyzed probably risk factors of AKI and in-hospital mortality. Independent-sample *t*-test or Chi-square test was performed to identify differences between AKI and non-AKI groups and survivors with AKI and non-survivors with AKI, respectively. The logistic regression model was applied to identify independent risk factors.

**Results:** AKI occurred in 71.94% of AAAD patients, including 85 stage 1 (35.26%), 77 stage 2 (31.95%), and 79 stage 3 (32.78%) patients. The in-hospital mortality rate was 21.16%. Logistic regression analysis showed that the body mass index, chronic kidney disease, chronic liver disease, cardiopulmonary bypass duration, red blood cell transfusion, and hypoproteinemia were the independent significant risk factors of the occurrence of post-operative AKI. The risk factors associated with in-hospital mortality among AAAD-induced AKI patients included AKI stage (odds ratio (OR), 3.322), deep hypothermic circulatory arrest (OR, 2.586), lactic acidosis (OR, 3.407), and continuous renal replacement therapy (OR, 3.156).

**Conclusion:** For AAAD patients undergoing surgery, AKI was a common complication, and it increased patients' mortality risk. Therefore, identifying the risk factors of AKI and preventing post-operative AKI are important for improving the post-operative outcomes of AAAD patients.

**Clinical Trial Registration:** ChiCTR, ChiCTR1900021290. Registered 12 February 2019, http://www.chictr.org.cn/showproj.aspx?proj=35795.

## Introduction

Acute kidney injury (AKI), which is mainly defined as a sudden onset of decreased renal function characterized by increased serum creatinine (SCr) levels or oliguria, is a common complication during perioperative stage and associated with prolonged hospitalization and high mortality. The incidence of AKI associated with cardiovascular surgery varies according to the type of operation, usually ~4.2–36.0% using either the risk, injury, failure, loss of kidney function, and end-stage kidney disease (RIFLE), the Acute Kidney Injury Network (AKIN), or the Kidney Disease Improving Global Outcomes (KDIGO) criteria (1–5). Cardiac surgery-associated acute kidney injury (CSA-AKI) is a widely recognized complication after cardiac surgery by cardiopulmonary bypass (CPB), and it is the second most common cause of AKI in the intensive care unit (6). Despite surgical advances, acute type A aortic dissection (AAAD) remains one of the most dangerous emergency in cardiac surgery, with high mortality and morbidity. The incidence of post-operative AKI in patients undergoing emergent operations for AAAD varies widely from 20.2 to 66.7% (7–9). The lack of a uniform definition for AKI is the main cause of the high variability, which has complicated studies in this field and has made comparisons of results difficult (10).

In 2012, the latest definition of AKI was proposed by the KDIGO Acute Kidney Injury Work Group. In combination with the RIFLE and AKIN criteria, the KDIGO criteria was designed to diagnose AKI earlier. According to the KDIGO criteria, AKI was defined as an increase in SCr by at least 0.3 mg/dl (≥26.4 μmol/L) within 48 h, an increase in SCr to ≥1.5 times baseline value, which is known or presumed to have occurred within the prior 7 days, or urine volume <0.5 ml/kg/h for 6 h. The KDIGO criteria tends to be more sensitive to detect AKI and predict in-hospital mortality (11). However, data on the incidence of AKI defined by the newest KDIGO criteria and in-hospital mortality of a homogeneous population who underwent acute aortic dissection are limited.

This study aimed to identify the incidence and prevalence of AKI defined by the KDIGO criteria during the whole hospital stay and the in-hospital mortality and to analyze the risk factors of the prognosis of AKI among AAAD patients. We also sought to clarify the incidence of AKI in a large population of patients who received surgical treatment for AAAD and to investigate its risk factors for the prognosis of AAAD-AKI patients.

## Methods

This is a monocenter retrospective study conducted at West China Hospital, Sichuan University, China. Ethical approval for this study was obtained from the ethical committee of our institution, and informed consent was waived because this was a retrospective study using clinical data.

### Patients

In this retrospective study, we included all the patients with AAAD for surgery under hypothermic circulatory arrest in our institution from March 2009 to June 2016. The exclusion criteria were as follows: (a) patients who experienced chronic dialysis within the past month or urgent pre-operative dialysis; (b) patients who had a history of kidney transplantation; and (c) patients with incomplete data. After screening the electronic medical records and laboratory test results, a total of 388 consecutive patients were collected according to the Stanford classification at the Institute of Cardiac Surgery, West China Hospital of Sichuan University.

### Data Collection

The detailed demographic, clinical, and laboratory data of the patients were collected from the electronic and paper medical records. Participants' demographics included age, sex, weight, height, and date of hospital admission and discharge. The main chronic medical conditions included hypertension/coronary heart disease (CHD), congestive heart failure, left ventricular ejection fraction of <35%, Marfan syndrome, chronic kidney disease (CKD), chronic obstructive pulmonary disease (COPD), diabetes, chronic liver disease, previous cardiac surgery, aortic regurgitation, hypercholesterolemia, and hypertriglyceridemia. Detailed comorbidity conditions consisted of systemic inflammatory response syndrome (SIRS), acute respiratory distress syndrome (ARDS), hepatic failure, hypoproteinemia, and lactic acidosis. The length of stay (LOS) in the hospital and laboratory data was also collected. Operation-related variables were the specific surgical procedure performed, the duration of CPB and aortic cross-clamping, and the use and duration of deep hypothermic circulatory arrest (DHCA) or moderate hypothermic circulatory arrest (MHCA).

### Definition

We used KDIGO definition as our diagnostic criteria for AKI, which reflected an abrupt (within 48 h) decline in renal function. AKI was defined as an absolute increase in SCr levels of ≥26.4 μmol/L within 48 h or ≥50% within 7 days or a reduction in urine output (documented oliguria of <0.5 ml/kg/h for >6 h). AKI stage 1 was defined as an increase in SCr levels of ≥26.4 μmol/L within 48 h or to 1.5–1.9 times of the baseline value within 7 days; AKI stage 2 was defined as an increase in SCr levels to 2.0–2.9 times of the baseline value; AKI stage-3 was defined as an increase in SCr levels to ≥3 times of the baseline value, an absolute increase in SCr levels of ≥354 μmol/L, or the initiation of renal replacement therapy (RRT). In our study, we applied the Cr criteria and initiation of RRT to diagnose AKI due to the lack of data on the 6- or 12-h urine volumes and usage of diuretics. We considered the lowest value of SCr measured within 2 days prior to hospital admission as the baseline SCr value. If baseline SCr was not available, we considered the first SCr value available within 2 days after admission as the baseline SCr values and compared the values with the subsequent levels for the following 2 days. Chronic liver disease was defined as one of the following conditions: chronic HBV infection, chronic HCV infection, alcoholic liver disease, non-alcoholic fatty liver disease, and liver cirrhosis due to any cause (12). Systemic inflammatory response syndrome (SIRS) was diagnosed if two of the following criteria are met: respiratory rate >20 breaths/min, heart rate >90 beats/min, leucocyte count >12,000 cells/μl or <4,000/μl, and body temperature >38 or <36°C (13). Acute respiratory distress syndrome (ARDS) was defined according to the Berlin definition (14). The definition of post-operative hepatic failure was “post-operative deterioration of synthetic, excretory, and detoxification functions of the liver, characterized by elevated INR and hyperbilirubinemia on, or after, post-operative day 5” (15). Lactic acidosis was defined as a serum lactate concentration of 5 mmol/L or greater and arterial blood pH <7.35 (16). LOS was defined as the number of days between the date of admission and the date of discharge or death. The other laboratory data in **Table 3** were obtained before the occurrence of AKI in the AKI group, whereas, in the non-AKI group, the data were obtained during the whole ICU stay. In our institution (from 2009 to 2016), all patients were cooled to a nasopharyngeal temperature of 20–24°C (DHCA) or 25–28°C (MHCA) in the aortic arch replacements for AAAD, with antegrade cerebral perfusion.

### Statistical Analysis

In the present study, patients were divided into the following groups according to the occurrences of AKI and in-hospital mortality: AKI vs. non-AKI group and survivors vs. non-survivors group, respectively. Continuous variables were described as mean ± standard deviations (standard deviation (SD), while categorical data were reported as absolute values and percentages. Comparisons of continuous variables between groups were performed using independent samples *t*-test or Wilcoxon rank sum test and comparisons of categorical variables between groups were performed using Chi-square test or Fisher's exact test. Differences between AKI-stage1, AKI-stage 2, and AKI-stage 3 groups were detected using one-way *ANOVA* test, and *LSD-t*-test was performed for multiple comparisons. Logistic regression analyses were used to identify the independent risk factors of AKI. Univariate analyze was firstly performed, and only those variables found statistically significant were used for stepwise multivariate logistic regression analyses. *P* < 0.05 indicated a statistical significance. Statistical analysis was performed using SPSS software version 21.0 (SPSS, Chicago, IL, USA).

## Results

### Characteristics of Patients With AAAD

A total of 335 patients were included in this retrospective analysis after excluding 53 patients (11 patients had chronic dialysis within the past month or were on dialysis before operation, 2 patients had a functioning kidney transplant, 7 patients died during or within 48 h after surgery, and 33 patients had incomplete data). The mean age of the patients with AAAD was 47.57 ± 10.31 years, and 80.60% were male (Table 1). The primary pre-operative factors, intraoperative factors, comorbidity, and laboratory data are summarized in Table 1. The mean CPB duration was 257.20 ± 57.90 min. MHCA was performed in 248 patients (74.03%), and DHCA was applied in 87 patients (25.97%). The baseline SCr level was 90.18 ± 54.46 μmol/L and the LOS in hospital was 22.54 ± 9.90 days. In total, 41 patients (12.24%) required continuous renal replacement therapy (CRRT) after the operation. The whole in-hospital mortality rate was 17.91%.

**Table 1:** Demographics and perioperative data of patients in this cohort.

Age (year; mean ± SD)	47.57 ± 10.31
Gender [*n* (%)]	
Male	270 (80.60)
Female	65 (19.40)
BMI (kg/m^2^)	24.86 ± 3.79
Preoperative factors [*n* (%)]	
Hypertension/CHD	181 (54.03)
Congestive heart failure	101 (30.15)
A left ventricular ejection fraction of <35%	27 (8.06)
Marfan syndrome	11 (3.28)
CKD	23 (6.87)
COPD	17 (5.07)
Diabetes	10 (2.99)
Chronic liver disease	15 (4.48)
Previous cardiac surgery	17 (5.07)
Aortic regurgitation	134 (40.00)
Hypercholesterolemia	20 (5.97)
Hypertriglyceridemia	82 (24.48)
Intraoperative factors	
MHCA [*n* (%)]	248 (74.03)
DHCA [*n* (%)]	87 (25.97)
CPB duration (min)	257.20 ± 57.90
Red blood cell transfusion (units)	3.04 ± 2.69
Lowest HGB intraoperative (g/L)	75.04 ± 10.77
Complication [*n* (%)]	
SIRS	109 (32.54)
ARDS	47 (14.03)
Hepatic failure	41 (12.24)
Hypoproteinemia	72 (21.49)
Lactic acidosis	62 (18.51)
Laboratory	
Baseline Scr (μmol/L)	90.18 ± 54.46
BUN (mmol/L)	7.46 ± 4.23
UA (umol/L)	368.51 ± 127.07
Cys-C (mg/L)	1.98 ± 1.53
HGB (g/L)	128.32 ± 19.96
Low hematocrit levels [<24%; *n* %)]	61 (18.21)
PLT (10^9^/L)	166.85 ± 95.89
WBC (10^9^/L)	11.42 ± 7.18
*N* (%)	79.65 ± 10.92
Surgical treatment	
Hemiarch reconstruction	125 (37.3)
Total arch replacement	210 (62.7)
LOS in hospital (days)	22.54 ± 9.90
CRRT [*n* (%)]	41 (12.24)
Mortality [*n* (%)]	60 (17.91)

*Continuous variables with normal distribution were reported as the mean ± standard deviation (SD); categorical variables were reported as number (percentage)*.

*SD, standard deviation; BMI, body mass index; CHD, coronary heart disease; CKD, chronic kidney disease; COPD, chronic obstructive pulmonary disease; MHCA, moderate hypothermic circulatory arrest; DHCA, deep hypothermic circulatory arrest; CPB, cardiopulmonary bypass; HGB, hemoglobin; SIRS, systemic inflammatory response syndrome; ARDS, acute respiratory distress syndrome; Scr, serum creatinine; BUN, blood urea nitrogen; UA, uric acid; Cys-C, cystatin C; PLT, platelet; WBC, white blood cell; N, neutrocyte; LOS, length of stay; CRRT, continuous renal replacement therapy*.

### Risk Factors for AKI in Patients With AAAD

AKI was present in 71.94% (241 patients) of patients, with 35.26, 31.95, and 32.78% of patients classified as having AKI stages 1, 2, and 3, respectively, according to the KDIGO criteria. The mean age of the patients with AKI was 47.95 ± 10.60 years, and the data comprised a male pre-dominance of 201 men (83.40%) and 40 women (16.60%) (Table 2).

**Table 2:** Sociodemographic, clinical, and laboratory data associated with non-AKI and AKI according to KDIGO criteria.

	**Non-AKI**	**AKI**	**Stage 1**	**Stage 2**	**Stage 3**	***P*-value^**a**^**	***P*-value^**b**^**
**Age (year; mean** **±** **SD)**	46.61 ± 9.51	47.95 ± 10.60	45.88 ± 10.10	49.29 ± 10.11	48.87 ± 11.36	0.284	0.079
**Gender [*****n*** **(%)]**							
Male	69 (73.40)	201 (83.40)	70 (82.35)	64 (83.12)	67 (84.81)	**0.038**	0.675
Female	25 (26.60)	40 (16.60)	15 (17.65)	13 (16.88)	12 (15.19)		
**BMI (kg/m**^**2**^**)**	23.59 ± 2.96	25.35 ± 3.97	25.23 ± 3.49	25.60 ± 3.47	25.25 ± 4.85	**0.000**	0.810
**Preoperative factors [*****n*** **(%)]**							
Hypertension/CHD	42 (44.68)	139 (57.68)	49 (57.65)	43 (55.84)	47 (59.49)	**0.032**	0.818
Congestive heart failure	24 (25.53)	77 (31.95)	27 (31.76)	19 (24.68)	31 (39.24)	0.250	0.323
A left ventricular ejection fraction of <35%	11 (11.70)	16 (6.64)	4 (4.71)	6 (7.79)	6 (7.59)	0.126	0.453
Marfan syndrome	3 (3.19)	8 (3.32)	2 (2.35)	3 (3.90)	3 (3.80)	1.000	0.670
CKD	1 (1.06)	22 (9.13)	10 (11.76)	5 (6.49)	7 (8.86)	**0.007**	0.506
COPD	2 (2.13)	15 (6.23)	3 (3.53)	5 (6.49)	7 (8.86)	0.168	0.198
Diabetes	3 (3.19)	7 (2.90)	1 (1.18)	1 (1.30)	5 (6.33)	1.000	0.063
Chronic liver disease	9 (9.57)	6 (2.49)	2 (2.35)	3 (3.90)	1 (1.27)	**0.014**	0.807
Previous cardiac surgery	9 (9.57)	8 (3.32)	5 (5.88)	1 (1.30)	2 (2.53)	**0.026**	0.282
Aortic regurgitation	33 (35.11)	101 (41.91)	37 (43.53)	31 (40.26)	33 (41.77)	0.254	0.814
Hypercholesterolemia	1 (1.06)	19 (7.88)	5 (5.88)	8 (10.39)	6 (7.59)	**0.018**	0.670
Hypertriglyceridemia	15 (15.96)	67 (27.80)	18 (21.18)	23 (29.87)	26 (32.91)	**0.024**	0.093
**Intraoperative factors**							
MHCA [*n* (%)]	64 (68.09)	184 (76.35)	68 (80.00)	61 (79.22)	55 (69.62)	0.121	0.122
DHCA [*n* (%)]	30 (31.91)	57 (23.65)	17 (20.00)	16 (20.78)	24 (30.38)	0.121	0.122
CPB duration (min)	241.00 ± 44.02	263.52 ± 61.41	252.66 ± 53.72	262.25 ± 64.95	276.46 ± 63.93^*^	**0.000**	**0.044**
Red blood cell transfusion (units)	2.30 ± 2.36	3.33 ± 2.76	3.01 ± 2.73	3.14 ± 2.61	3.84 ± 2.89	**0.013**	0.128
Lowest HGB intraoperative (g/L)	75.75 ± 10.21	74.77 ± 10.99	76.01 ± 10.47	73.80 ± 11.88	74.37 ± 10.63	0.455	0.411
**Complication [*****n*** **(%)]**							
SIRS	26 (27.66)	83 (34.44)	27 (31.76)	21 (27.27)	35 (44.30)	0.234	0.099
ARDS	16 (17.02)	31 (12.86)	9 (10.59)	11 (14.29)	11 (13.92)	0.325	0.519
Hepatic failure	17 (18.09)	24 (9.96)	5 (5.89)	10 (12.99)	9 (11.39)	**0.041**	0.231
Hypoproteinemia	5 (5.32)	67 (27.80)	34 (40.00)	12 (15.58)	21 (26.58)	**0.000**	**0.049**
Lactic acidosis	13 (13.83)	49 (20.33)	12 (14.12)	14 (18.18)	23 (29.11)	0.169	**0.018**
**Laboratory**							
Baseline Scr (μmol/L)	84.16 ± 37.98	92.54 ± 59.59	80.77 ± 27.20	81.16 ± 38.86	116.16 ± 88.21^*^▴	0.206	**0.000**
BUN (mmol/L)	6.96 ± 3.65	7.66 ± 4.43	6.84 ± 3.30	7.43 ± 4.77	8.78 ± 4.93^*^	0.174	**0.016**
UA (μmol/L)	358.85 ± 139.59	372.27 ± 121.94	342.33 ± 114.81	376.56 ± 111.50	400.31 ± 132.87^*^	0.386	**0.009**
HGB (g/L)	130.90 ± 20.30	127.32 ± 19.78	128.44 ± 19.22	130.03 ± 18.97	123.47 ± 20.77	0.139	0.095
Low hematocrit levels [<24%; *n* (%)]	8(8.51)	43(17.84)	14 (16.47)	13 (16.88)	16 (20.25)	**0.033**	0.790
PLT (10^9^/L)	171.11 ± 80.66	161.46 ± 85.47	184.22 ± 96.50	170.05 ± 92.34	128.59 ± 49.06^*^	0.613	**0.000**
WBC (10^9^/L)	11.30 ± 11.88	11.47 ± 4.14	11.09 ± 4.26	11.67 ± 3.88	11.67 ± 4.26	0.897	0.585
*N* (%)	78.00 ± 10.83	80.30 ± 10.91	79.14 ± 9.72	80.24 ± 11.90	81.60 ± 11.11	0.084	0.355
LOS in hospital (days)	21.71 ± 8.98	22.86 ± 10.23	22.48 ± 7.53	25.03 ± 12.41	21.18 ± 10.21	0.342	0.059

*Continuous variables with normal distribution were reported as the mean ± standard deviation (SD); categorical variables were reported as number (percentage). Student's t-test was used to compare means of two continuous normally distributed variables, and Chi-square test or Fisher's exact test was used for categorical variables*.

a *AKI vs. non-AKI*.

b *Among AKI stage 1, stage 2, and stage 3*.

* *AKI stage 3 vs. AKI stage 1. The difference is statistically significant; ▴AKI stage 3 vs. AKI stage 2. The difference is statistically significant*.

*AKI, acute kidney injury; KDIGO, Kidney Disease: Improving Global Outcomes; SD, standard deviation; BMI, body mass index; CHD, coronary heart disease; CKD, chronic kidney disease; COPD, chronic obstructive pulmonary disease; MHCA, moderate hypothermic circulatory arrest; DHCA, deep hypothermic circulatory arrest; CPB, cardiopulmonary bypass; HGB, hemoglobin; SIRS, systemic inflammatory response syndrome; ARDS, acute respiratory distress syndrome; Scr, serum creatinine; BUN, blood urea nitrogen; UA, uric acid; Cys-C, cystatin C; PLT, platelet; WBC, white blood cell; N, neutrocyte; LOS, length of stay. The bold values means they are <0.05, which is statistically significant*.

As shown in Table 2, the distribution of pre-operative factors, intraoperative factors, complication, and laboratory data of the patients is presented according to the AKI stages and occurrence of AKI or not. In the univariate analysis of risk associated with AKI for patients with AAAD, a significance difference was found in sex, body mass index (BMI), hypertension/CHD, CKD, chronic liver disease, previous cardiac surgery, hypercholesterolemia, and hypertriglyceridemia between the non-AKI and AKI groups (*P* < 0.05). Regarding the intraoperative factors, the AKI group had significantly higher CPB duration and red blood cell transfusion than the non-AKI group (*P* < 0.05). The proportion of patients with hepatic failure, hypoproteinemia, and low hematocrit levels (<24%) were significantly higher in the AKI group than in the non-AKI group. Among patients with AKI stages 1, 2, and 3, CPB duration, hypoproteinemia, lactic acidosis, baseline Scr, BUN, UA, and PLT were significantly different (*P* < 0.05, Table 2). However, none of pre-operative factors showed any significant differences among all AKI stages. The results of the multivariate analysis for AKI predictors are summarized in Table 3. For patients with AAAD, the independent risk factors for AKI included BMI (OR, 1.221; 95% confidence interval (CI), 1.148–1.245), CKD (OR, 12.352; 95% CI, 1.207–126.414), chronic liver disease (OR, 2.207; 95% CI, 1.452–5.643), CPB duration (OR, 1.008; 95% CI, 1.002–1.013), red blood cell transfusion (OR, 1.186; 95% CI, 1.055–1.334), and hypoproteinemia (OR, 5.091; 95% CI, 1.855–13.976) (Table 3).

**Table 3:** Multivariate analysis of risk factors associated with AKI.

**Variables**	**β**	**Odds ratio**	**95% confidence interval**		***P*-value**
			**Lower**	**Upper**	
BMI	0.200	1.221	1.148	1.245	0.001
CKD	2.514	12.352	1.207	126.414	0.034
Chronic liver disease	0.358	2.207	1.452	5.643	0.043
CPB duration	0.008	1.008	1.002	1.013	0.006
Red blood cell transfusion	0.171	1.186	1.055	1.334	0.004
Hypoproteinemia	1.627	5.091	1.855	13.976	0.002

*The odds ratio and 95% confidence interval were measured through binary logistic regression*.

*AKI, acute kidney injury; BMI, body mass index; CKD, chronic kidney disease; CPB, cardiopulmonary bypass*.

### Risk Factors for the Prognosis of AAAD-AKI

Table 4 lists the data of both survivors and non-survivors, which consisted of 190 (78.83%) and 51 (21.16%) patients, respectively. Univariate analysis was performed in the AAAD-AKI patients, and the AKI stage was found to be a significant risk factor. In the categorical variables, the proportion of patients with diabetes and DHCA were significantly higher in the non-survivors than in the survivors, and the proportion of patients undergoing MHCA was significantly lower in the non-survivors than in the survivors. In the continuous variables, the Cys-C value and CPB duration were higher in the non-survivors than in the survivors (2.95 ± 1.79 vs. 2.20 ± 1.55 mg/L and 282.98 ± 82.59 vs. 258.30 ± 53.43 min, *P* < 0.05). The LOS in hospital was longer in survivors than in non-survivors (24.63 ± 9.90 vs. 16.18 ± 8.52 days; *P* = 0.000). Among the patients with AKI stage 3, 41 patients (51.90%) received CRRT. Compared with the patients without CRRT, those treated with CRRT showed worse hospital outcomes (39.22 vs. 11.05%, *P* = 0.000) (Table 4).

**Table 4:** Univariate analysis of risk factors of in-hospital mortality for patients with AKI.

	**Non-survivors (*n* = 51)**	**Survivors (*n* = 190)**	***P*-value**
**Age (year; mean** **±** **SD)**	50.53 ± 10.69	47.26 ± 10.50	0.050
**Gender [*****n*** **(%)]**			
Male	46 (90.20)	155 (81.58)	0.142
Female	5 (9.80)	35 (18.42)	
**BMI (kg/m**^**2**^**)**	25.25.25 ± 5.43	25.38 ± 3.49	0.875
**AKI classification [*****n*** **(%)]**			
Stage 1	4 (7.84)	81 (42.63)	0.000
Stage 2	11 (21.57)	66 (34.74)	0.000
Stage 3	36 (70.59)	43 (22.63)	0.000
**Preoperative factors [*****n*** **(%)]**			
Hypertension/CHD	28 (54.90)	111 (58.42)	0.652
Congestive heart failure	20 (39.22)	57 (30.00)	0.210
A left ventricular ejection fraction of <35%	5 (9.80)	11 (5.79)	0.342
Marfan syndrome	3 (5.88)	5 (2.63)	0.371
CKD	3 (5.88)	19 (10.00)	0.583
COPD	5 (9.80)	10 (5.26)	0.323
Diabetes	4 (7.84)	3 (1.58)	0.038
Chronic liver disease	0 (0.00)	6 (3.16)	0.347
Previous cardiac surgery	2 (3.92)	6 (3.16)	0.678
Aortic regurgitation	16 (31.37)	86 (45.26)	0.075
Hypercholesterolemia	4 (7.84)	15 (7.89)	1.000
Hypertriglyceridemia	18 (35.29)	49 (25.79)	0.179
**Intraoperative factors**			
MHCA [*n* (%)]	32 (62.76)	152 (80.00)	0.010
DHCA [*n* (%)]	19 (37.25)	38 (20.00)	0.010
CPB duration (min)	282.98 ± 82.59	258.30 ± 53.43	0.047
Red blood cell transfusion (units)	3.89 ± 3.39	3.14 ± 2.56	0.144
Lowest HGB intraoperative (g/L)	76.01 ± 10.36	74.44 ± 11.15	0.365
**Complication [*****n*** **(%)]**			
SIRS	19 (37.25)	64 (33.68)	0.634
ARDS	7 (13.73)	24 (12.63)	0.836
Hepatic failure	6 (11.76)	18 (9.47)	0.628
Hypoproteinemia	10 (19.61)	57 (30.00)	0.141
Lactic acidosis	21 (41.18)	28 (14.73)	0.000
**Laboratory**			
Baseline Scr (μmol/L)	96.69 ± 50.90	91.35 ± 61.65	0.570
BUN (mmol/L)	7.45 ± 3.02	7.72 ± 4.74	0.698
UA (μmol/L)	390.25 ± 126.19	367.45 ± 120.66	0.237
Cys-C (mg/L)	2.95 ± 1.79	2.20 ± 1.55	0.003
HGB (g/L)	131.16 ± 19.54	126.28 ± 19.76	0.118
Low hematocrit levels [<24%; *n* (%)]	7 (13.73)	36 (18.95)	0.387
PLT (10^9^/L)	141.20 ± 62.47	166.90 ± 90.02	0.020
WBC (10^9^/L)	11.39 ± 3.98	11.49 ± 4.19	0.877
*N* (%)	81.16 ± 11.63	80.07 ± 10.73	0.527
LOS in hospital (days)	24.63 ± 9.90	16.18 ± 8.52	0.000
CRRT [*n* (%)]	20 (39.22)	21 (11.05)	0.000

*Continuous variables with normal distribution were reported as the mean ± standard deviation (SD); categorical variables were reported as number (percentage). Student's t-test was used to compare means of two continuous normally distributed variables, and Chi-square test or Fisher's exact test was used for categorical variables*.

*AKI, acute kidney injury; AAAD, acute type A aortic dissection; SD, standard deviation; BMI, body mass index; CHD, coronary heart disease; CKD, chronic kidney disease; COPD, chronic obstructive pulmonary disease; MHCA, moderate hypothermic circulatory arrest; DHCA, deep hypothermic circulatory arrest; CPB, cardiopulmonary bypass; HGB, hemoglobin; SIRS, systemic inflammatory response syndrome; ARDS, acute respiratory distress syndrome; Scr, serum creatinine; BUN, blood urea nitrogen; UA, uric acid; Cys-C, cystatin C; PLT, platelet; WBC, white blood cell; N, neutrocyte; LOS, length of stay; CRRT, continuous renal replacement therapy*.

Statistically significant risk factors were analyzed by stepwise multivariate logistic regression. For patients with AKI, independent risk factors for in-hospital mortality included the AKI stage (OR, 3.322; 95% CI, 1.816–6.077), DHCA (OR, 2.586; 95% CI, 1.131–5.916), lactic acidosis (OR, 3.407; 95% CI, 1.480–7.839), and CRRT (OR, 3.156; 95% CI, 1.278–7.795) (Table 5).

**Table 5:** Multivariate analysis of risk factors of in-hospital mortality for patients with AKI.

**Variables**	**β**	**Odds ratio**	**95% confidence interval**		***P*-value**
			**Lower**	**Upper**	
AKI stage	1.201	3.322	1.816	6.077	0.000
DHCA	0.950	2.586	1.131	5.916	0.024
Lactic acidosis	1.226	3.407	1.480	7.839	0.004
CRRT	1.149	3.156	1.278	7.795	0.013

*The odds ratio and 95% confidence interval were measured through binary logistic regression*.

*AKI, acute kidney injury; DHCA, deep hypothermic circulatory arrest; CRRT, continuous renal replacement therapy*.

## Discussion

A number of factors may contribute to AKI following cardiac surgery, including metabolic abnormalities, exogenous and endogenous toxins, necrohormone activation, ischemia and reperfusion injury, oxidative stress, and inflammatory response (17, 18). Numerous factors were identified in recent multicenter studies, including the pre-operative, intraoperative, and post-operative pathways, which probably play a role in the development of AKI (19–21). Moreover, the multiple risk factors, including age, overweight, AKI classification, renal failure, COPD, lactic acidosis, previous cardiac surgery, CPB duration, pre-operative SCr level, red blood cell transfusion, and lower body ischemic time, were reported to affect the occurrence and prognosis of AKI, which is related to increased mortality in AAAD patients (7–9, 22–26).

This retrospective study determined the incidence and risk factors of AKI, defined by KDIGO criteria, after the surgical treatment for AAAD. A total of 241 (71.9%) patients developed post-operative AKI, which, to the best of our knowledge, is the highest incidence of AAAD-AKI reported in the literature. In a previous study involving 403 Japanese patients, 181 (44.9%) experienced post-operative AKI, defined by the KDIGO criteria, after the operation for AAAD (8). A higher incidence of AKI was diagnosed in cardiac surgery based on the KDIGO criteria than on the RIFLE and AKIN criteria (11, 27). However, no study has compared the different definitions of AAAD-AKI. Zhao et al. (9) have reported an incidence of AKI, defined by the AKIN criteria, after aortic surgery of 66.7%, which is similar to our result. However, this retrospective study only included 108 patients with obesity. The much higher than described incidence of AKI in AAAD could be explained by several factors. First, many patients met the expansion of the time limit for percentage increase (≥50% baseline) in the KDIGO criteria from 48 h to 7 days. Second, the study population varied among published reports. Finally, the study cohort was presumably heterogeneous in terms of cohort and surgical status.

Several studies have reported the risk factors for post-operative AKI (28–30). We also retrospectively reviewed these factors in our study; however, most factors were not significant in the multivariate analysis. Regarding the intraoperative factors, especially CPB duration, MHCA, and DHCA, only CPB duration was significantly higher in the AKI group than in the non-AKI group in the univariate analysis. However, it was not statistically significant in the multivariate analysis. In the Korean cohort, they reported that prolonged CPB duration was an independent risk factor for AKI defined by the RIFLE criteria, but MHCA was not (23). In a recent study, Amano et al. (31) reviewed 191 patients who underwent AAAD and concluded that shorter body ischemic time should be recognized specifically as a modifiable surgical risk factor for post-operative AKI defined by the AKIN criteria. The renal medullary ischemia or reperfusion injury may be the most important pathophysiological change. The shorter lower body ischemic time may result in better renal and survival outcomes (31). The inconsistency of these results may be due to the different populations and inconsistent definitions and diagnoses of AKI.

The logistic regression analysis identified higher BMI (OR, 1.221; *P* = 0.001) was associated with increased AKI compared with the reference value. Previous reports have suggested that higher BMI was a dependent risk factor of AKI in AAAD patients (22, 31). Thus, AKI may occur in overweight patients (BMI ≥24 kg/m^2^), and a retrospective study was designed to investigate the incidence and risk factors of AKI in overweight patients only (9). Furthermore, our results demonstrated that patients with a history of CKD have a significantly higher risk for developing AKI after surgery for AAAD. From our perspective, it could be interpreted as “acute on chronic kidney disease” (A/C), which was also in consistence with some other studies (31, 32). In addition, the units of red blood cell transfusion in our study were higher in patients with AKI than in patients without AKI. Previous studies have also found red blood cell transfusion to be an independent risk factor for post-operative AKI (8, 23). In a recent randomized clinical trial, Garg et al. (33) demonstrated that a restrictive transfusion approach can reduce red blood cell transfusions in cardiac surgery without increasing the risk of AKI compared with a liberal transfusion approach. Although the causal relationship between red blood cell transfusion and AKI was not directly established in this relatively large-sample research, it seems that unnecessary red blood cell transfusions do not confer any additional benefit.

The overall post-operative in-hospital mortality rate was 21.2 and 9.6% in the AKI and non-AKI groups, respectively, and it increased with each stratification of the KDIGO criteria (stage 1, 7.84%; stage 2, 21.57%; stage 3, 70.59%). In patients with AAAD, the AKI group had a significantly higher in-hospital mortality rate than non-AKI group during hospital stay (21.16 vs. 9.57%; *P* < 0.001). The mortality rate in the AKI stage 3 group is the highest at 70.59% compared with the AKI stages 1 and 2 groups, and more advanced KDIGO classifications are associated with higher rates of mortality, in an approximately linear fashion. These results are consistent with the findings of some published reports (23, 34). In addition, AKI stage 3 has been reported to be significantly associated with reduced long-term survival after the operation for AAAD (8).

Two previous studies have reported that hypercalcemia in AAAD indicates an increased risk of post-operative mortality (26, 35). They considered that lactic acidosis, as a surrogate for systemic malperfusion, represents a novel, accurate, and easily obtainable predictor to estimate mortality. Therefore, we included lactic acidosis as a risk factor in the analysis and found that it was also an independent risk factor for prognosis in our AAAD patients.

Regarding the risk factors associated with hospital mortality for AAAD patients with AKI, the CPB duration, MHCA, and DHCA were found to be significant in the univariate analysis; however, only DHCA was found as an independent risk factor associated with mortality, indicating that the innovation of surgical strategies may be critical for improving the prognosis. Novel technologies reducing the duration of circulatory arrest, such as aortic balloon occlusion (36) and retrograde inferior vena caval perfusion (37), should be advocated due to their potential of attenuating ischemia reperfusion injury of vital organs of the lower body.

To date, in the absence of a specific therapy for post-operative AKI in AAAD patients, prevention strategies such as tight management of intraoperative hypotension and anemia, using novel technologies to reduce the duration of organ ischemia, avoidance of nephrotoxic exposure, may play an important role in renal function protection (38). More randomized controlled trials are needed to identify appropriate strategies to improve the prognosis of patients undergoing surgery for AAAD.

## Limitations

Some potential limitations should be recognized. First, since baseline SCr was taken after presentation with AAAD in part of the patients who might have experienced AKI pre-operatively, the incidence of AKI should be interpreted with caution. Second, this is a monocenter study; a number of patients included were mainly from Western China. Third, due to lack of urine output data, the incidence of AKI might even be underreported. Finally, the long-term outcomes were not collected, and follow-up of all patients should be underway.

## Conclusion

Although advances in surgical treatment for AAAD improved patients' outcomes, the morbidity of post-operative AKI is high, which is closely related to in-hospital mortality. BMI, CKD, chronic liver disease, CPB duration, red blood cell transfusion, and hypoproteinemia were found to be independent risk factors for post-operative AKI, whereas, AKI stage, DHCA, lactic acidosis, and LOS in hospital are significant risk factors associated with in-hospital mortality in AAAD-AKI patients. More clinical trials are needed to determine whether prevention strategies can reduce the incidence and mortality of AKI and economize resource utilization after the operation for AAAD.

## Data Availability Statement

The raw data supporting the conclusions of this article will be made available by the authors, without undue reservation.

## Ethics Statement

The studies involving human participants were reviewed and approved by the ethical committee of West China Hospital, Sichuan University. The ethics committee waived the requirement of written informed consent for participation.

## Author Contributions

LL, JZ, and TZ: conception and design, manuscript writing and editing. JZ and TZ: administrative support. LL, JZ, XH, and WZ: collection and assembly of data. LL, XH, DY, and YX: data analysis and interpretation. All authors read and approved the final manuscript.

## Conflict of Interest

The authors declare that the research was conducted in the absence of any commercial or financial relationships that could be construed as a potential conflict of interest.

## Abbreviations

AKI: acute kidney injury
AAAD: acute type A aortic dissection
KDIGO: Kidney Disease: Improving Global Outcomes
OR: odds ratio
SCr: serum creatinine
CSA-AKI: cardiac surgery-associated acute kidney injury
SD: standard deviation
BMI: body mass index
CHD: coronary heart disease
CKD: chronic kidney disease
COPD: chronic obstructive pulmonary disease
MHCA: moderate hypothermic circulatory arrest
DHCA: deep hypothermic circulatory arrest
CPB: cardiopulmonary bypass
HGB: hemoglobin
SIRS: systemic inflammatory response syndrome
ARDS: acute respiratory distress syndrome
BUN: blood urea nitrogen
UA: uric acid
Cys-C: cystatin C
PLT: platelet
WBC: white blood cell
N: neutrocyte
LOS: length of stay
CRRT: continuous renal replacement therapy.
